# Therapists and patients perceptions of a mixed reality system designed to improve trunk control and upper extremity function

**DOI:** 10.1038/s41598-024-55692-4

**Published:** 2024-03-19

**Authors:** M. Scheermesser, D. Baumgartner, I. Nast, J. Bansi, J. Kool, P. Bischof, C. M. Bauer

**Affiliations:** 1 School of Health Sciences, Institute of Physiotherapy, Zurich University of Applied Sciences, Katharina-Sulzer-Platz 9, 8401 Winterthur, Switzerland; 2 School of Engineering, Institute of Mechanical Systems IMES, Zurich University of Applied Sciences, Technikumstrasse 71, 8400 Winterthur, Switzerland; 3 Kliniken-Valens, Research and Development, Rehabilitation Centre Valens, Taminaplatz 1, 7317 Valens, Switzerland; 4 Department of Health, Physiotherapy, OST-University of Applied Sciences Eastern Switzerland, Rosenbergstrasse 59, 9001 St. Gallen, Switzerland; 5 Lake Lucerne Institute, Seestrasse 18, 6354 Vitznau, Switzerland; 6 Faculty of Sport and Health Science, University of Jyväskylä, PO Box 35, 40014 Jyvaskyla, Finland

**Keywords:** Medical devices and technologies, Augmented reality, Trunk control, Rehabilitation, Stroke, Stroke, Biomedical engineering

## Abstract

A prototype system aimed at improving arm function and trunk control after stroke has been developed that combines mixed-reality (MR) feedback with a mobile seat system (Holoreach). The purpose of this study was to assess the usability of Holoreach in a rehabilitation setting from both the patient and therapist perspective. Ten therapists (eight physiotherapists and two occupational therapists) used the device in their regular therapy programs for fifteen stroke patients with trunk control issues. Each patient received four individual therapy sessions with the device performed under the supervision of the therapist. Therapists and patients kept therapy diaries and used customized questionnaires. At the end of the study two focus groups were conducted to further assess usability. Generally, the prototype system is suitable for training trunk and arm control. The therapists expressed overall positive views on the impact of Holoreach. They characterized it as new, motivating, fresh, joyful, interesting, and exciting. All therapists and 80% of the patients agreed with the statement that training with Holoreach is beneficial for rehabilitation. Nonetheless, improvements are required in the hardware and software, and design. The prototype system contributes at various levels to the rapidly evolving advances in neurorehabilitation, particularly regarding the practical aspect of exercise delivery.

## Introduction

Early post-stroke impairment of trunk control is a significant cause of limited mobility and characterized by the inability of the trunk musculoskeletal system to maintain the body in an upright position, adjust to perturbations or weight shifts, or control trunk movement^1,2^. Reduced trunk control is associated with limitations in breathing, speech, balance, gait, arm, and hand function^1–5^. Additionally, sitting balance is an important predictor of motor and functional recovery after a stroke^2–5^. Trunk control has been identified as a prerequisite in regaining the ability to stand and walk, and an important predictor of the total functional outcome of the rehabilitation, particularly regarding standing and locomotion^6,7^. Severity of disability and extend of recovery early post stroke are important predictors of outcome at six months post stroke. As the most significant improvements typically occur in the early post stroke phase, especially motor recovery, it is important to start therapy early^6–8^. Trunk control can be quantified with clinical measurement scales such as the trunk control test or the trunk impairment scale^9,10^, or through objective methods such as 3D kinematic measures or wearables^11,12^. Trunk control exercises are effective in improving trunk control, balance, and mobility after stroke^13^. Studies show that patients whose trunk control improves faster will be able to start earlier with gait and balance training^14^. Exercises that incorporate reaching beyond arm’s length during sitting yield a positive effect on gait and mobility related functions and abilities^15^. Moreover, there is a significant correlation between the Berg Balance Scale, assessed at discharge, and trunk muscle strength^16^. Nevertheless, only a few studies investigate trunk control rehabilitation^13,15^. Traditional trunk control rehabilitation is resource intensive regarding time and physical resources implying that the critical threshold for improvement might not be reached^17,18^. Rehabilitation technologies are an effective add-on for conventional therapy to improve semi-independent training at a higher intensity^19^. Thus, further innovation is desirable to augment trunk control rehabilitation in the early phase post stroke aiming at providing high dose trunk control training^14,15,20,21^. Mindful of this gap, we developed a prototype of an assisted therapy chair that produces exercise stimuli for trunk control training, standing, and walking in early post stroke^22^. This device has shown good usability and effectiveness as an adjunct to conventional trunk control rehabilitation^22,23^. In this study, the chair is enhanced with a Mixed Reality (MR) application (smart glasses) to train reaching beyond the participant’s arm length (Holoreach). The Holoreach development process followed a User Centered Design (UCD) approach in which potential users were involved right from the outset of development of the technology. This was to ensure that the structure, content, and design of the technology is driven by the needs, expectations, and understanding of the users. The UCD approach helps developers to identify and fulfil user needs and requirements at the prototype stage of a technology^24^.

The aim of this study was to test the Holoreach during inpatient rehabilitation with both therapists and post stroke patients to assess their needs and requirements. The results of this study will form the basis for the development of future prototype generations.

## Methods

### Participants

Ten therapists from an inpatient rehabilitation clinic participated in the study. Therapists recruited 15 patients after stroke who had been assessed following site specific standard operating procedures and guidelines. The therapists were taken on in the study if they were working in stroke rehabilitation at the time of the study. Inclusion criteria for the patients were a primary diagnosis of stroke, age ≥ 18 years, the ability to sit independently for at least 10 s, a Trunk Impairment Scale (TIS) score between 2 and 19 points^10^, the ability to understand and follow verbal instructions, and the ability to perform therapy for at least 2 h per day. Exclusion criteria were existing or previous spinal or upper limb disorders which would hamper participation in the exercise or an MoCA score < 3^25^. The TIS spanned a wide range to cover the full spectrum of potential future Holoreach users.

### Devices

**Figure 1:**
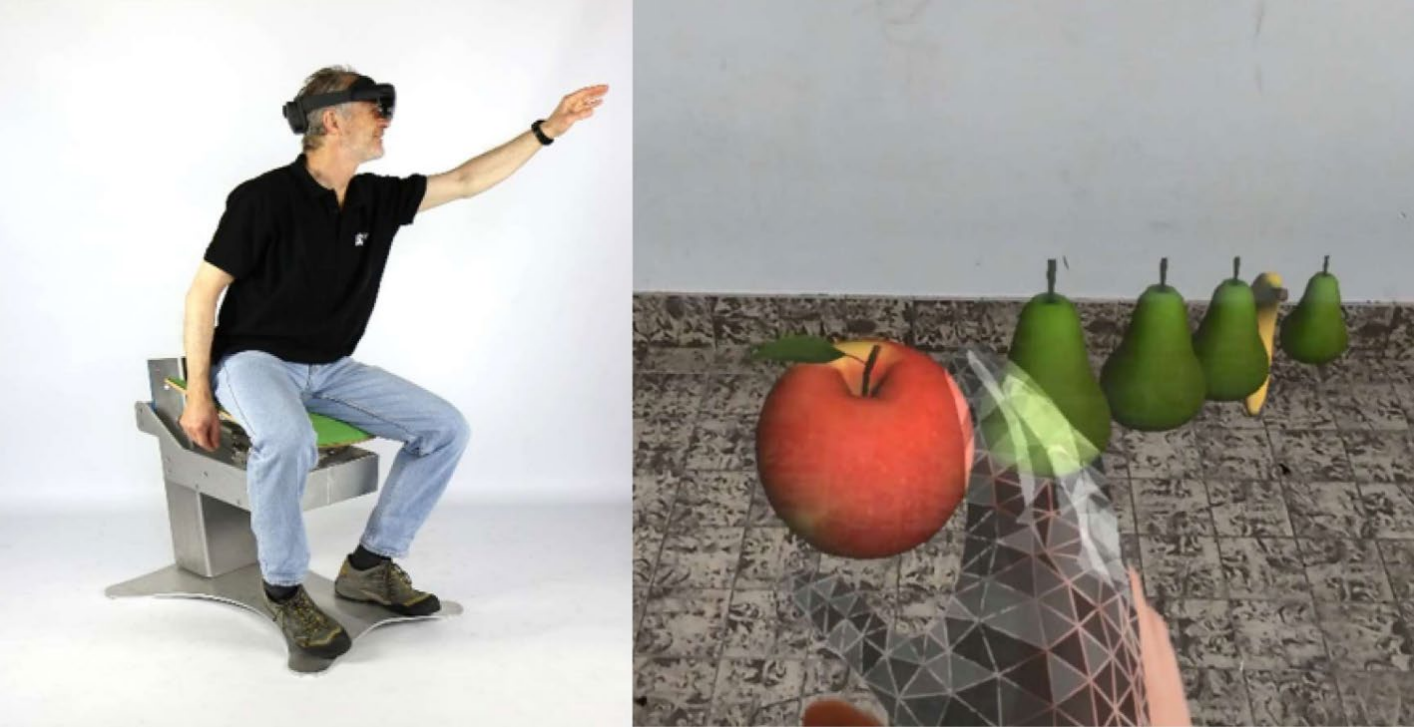
A reaching movement and the visualization of the objects and the hand during the Holoreach exercise (notebook not shown).

The MR device comprises an assistive therapy chair^22^, a computerized system based on the MR Microsoft® HoloLens II (MHL) and a notebook to monitor the patient’s view (Fig. 1)^26^.

The assistive therapy chair has been described in detail elsewhere and, therefore, is summarized only briefly here^22,23,27–29^. The seat was designed for the rehabilitation of trunk control in patients after stroke and has shown good usability and effectiveness as an adjunct to conventional trunk control rehabilitation^22,23^. Patients training on the device improved beyond the clinically minimal important difference for trunk control and gait speed^23^.

The MHL involves a pair of MR smart glasses to generate an environment in which real and virtual elements appear to coexist. In contrast to augmented reality, which overlays digital information onto the user's view of the real world, enhancing their perception and interaction with their immediate environment, MR integrates and anchors virtual content into the user's physical surroundings, allowing for dynamic interactions and coexistence of both real and virtual elements in a unified environment^30,31^. With the MR-based head-mounted display the user becomes the protagonist in an immersive experience that allows the user to interact with the surrounding environment using holograms whilst engaging their senses throughout^32^. The requirements on the MR application were based on personas portraying the prototypical users. These personas were developed following a conceptualization phase that involved therapists through work shadowing, interviews, and observations including thinking out loud techniques.

### Procedure

The therapists received 3 h of instruction by the investigators prior to the start of this study. The therapists integrated the Holoreach into a rehabilitation setting as part of the usual physiotherapy program with a total duration of 9.5 h per week. In addition, Holoreach training sessions that focused on improving mobility and trunk control took place for 5 h per week. Occupational therapy accounted for 3 h per week and was aimed at improving cognitive functioning in daily activities, such as washing and dressing in the morning, and household activities. Other treatments (1.5 h per week) were scheduled as needed based on the individual patient’s needs. These included robotic and medical exercise training, neuropsychological training, counselling by a social worker, and recreational therapy. The same therapist who adjusted the chair settings supervised all sessions of a participant. If required, the therapists assisted the patient’s transfer to the chair and secured them on the chair.

#### Exercise game

An interactive game was designed for a standard notebook to be played in a normally illuminated room. The game was developed together with therapists. Moving fruits (bananas, apples, and pears) are displayed by means of holograms in a 3D-environment. The height of the fruit varies, as does the reaching distance to them and their circular placement around the patient. A red arrow—present as a hologram—aids the patient in finding the position of the fruit. If the hand of the patient overlaps the 3D-placement of the fruit for more than 1 s, the fruit disappears, auditory feedback^33^ is provided immediately and another fruit appears elsewhere in the 3D-environment. An algorithm automatically calculates the patient’s success rate and adapts the reaching distance accordingly. The success rate is displayed at the end of each exercise to provide delayed explicit feedback. Fruit also makes an appearance outside the immediate field of view of the MHL in order to stimulate rotational motion of the head and trunk to find the fruit. The game is made more difficult by combining it with increased levels of instability of the mobile seat so trunk control and sitting balance have to be maintained during the reaching exercises. By combining upper extremity reaching with considerable trunk mobilization of flexion/extension, lateral bending in various combinations, a substantial kinematic chain of human motion can be activated^27,34,35^. The game was developed in line with current advanced upper extremity function and trunk control rehabilitation and consideration of the importance of these functions for activities of daily living (ADL)^23,36^. The starting position for the patients is to sit as upright as possible with their head facing forwards, similarly to an ergonomic work position^22^.

### Data collection

Details of the data collection method are given in the Supplementary file. Participants were selected according to the purposive sampling method^37^ based on the intention of this study. We hypothesized that each participant would be providing unique information valuable to this study. Because of this, members of the accessible sample are not interchangeable, and the sample size was determined by data saturation and not statistical power analysis^37^.

Therapists: A mixed-method approach was used to explore the usability of Holoreach from the therapist perspective: keeping a diary, using customized questionnaires, general feedback and focus group interviews. The therapists completed a diary entry for each patient after each treatment session detailing aspects of preparation, training time for the affected and non-affected arm, treatment goals, instructions given to the patient, reasons for early termination of the exercise session (if applicable), the score the patient reached during the exercise, and the occurrence of adverse events. At the end of the study, the general views of the therapists regarding handling were captured by means of a survey (preparation 8 questions; calibration 5 questions), the exercise (6 questions), overall training (4 questions) and safety (4 questions) with a six-point Likert scale (“1—strongly disagree”—“6 strongly agree”). Furthermore, the therapists responded to three open questions: any problems they had encountered, their overall views, and their preferences for the focus group themes. Finally, focus group interviews were conducted to learn more about the personal experiences of each therapist with the Holoreach. Two focus group interviews were conducted. Both focus group sessions lasted 1 h and were audio recorded. The audio recordings were subsequently transcribed, both chronologically and in summary. The statements were thereby translated from colloquial German into formal German. The research team devised the main categories for the interview guidelines based on the research questions and the aim of the study. The guidelines for the focus group discussions covered the topics of “current physical therapy practice in stroke rehabilitation”, “general impressions”, “preparation”, “functionality”, “therapy with Holoreach”, “dents” and “concluding remarks”.

Stroke patients: The patients completed a diary entry after each training session that recorded perceived exhaustion following training of the legs, trunk, affected and non-affected arm (11-point Likert scale 0 “not exhausting at all”—10 “very exhausting”), as well as the occurrence of adverse events. At the end of the study, the general views of the patients on preparation (7 questions), calibration (5 questions), the exercise (7 questions), overall training (4 questions) and safety (4 questions) (the same Likert scale as above) were captured with a custom-made questionnaire. Furthermore, the patients responded to two open questions: the problems they encountered and their overall impression.

### Data analyses

Quantitative data from diaries and questionnaires were analyzed descriptively using IBM SPSS Statistics version 26 and Microsoft 365 Excel. Response frequencies, means, standard deviations, ranges and interquartile ranges were calculated. The focus group sessions were analyzed for themes using content structuring analysis^38^. The usability of a medical device has multiple aspects and in this study the focus was on effectiveness, efficiency, satisfaction, and safety^39^. A triangulation approach was chosen to obtain a multi-layered understanding of the usability of Holoreach^40,41^. Triangulation helps consider and record diversity and contradictions of a research object. Different methodological procedures and different data are converged (between-method) to uncover emerging themes. This complementary mixed-method approach enhances the strengths and minimizes the weaknesses of a mono-method approach^42^. Quantitative and qualitative data were integrated according to the triangulation method^40,41^.

### Ethical approval and consent to participate

All the methods were performed in accordance with relevant guidelines and regulations. The Ethics Committee of Eastern Switzerland (EKOS), Switzerland, verified the study juristically (Req-2020-00564). Informed consent was obtained from all the participants to participate in the study. All participants gave their written informed consent for their data to be published.

## Results

### Participants

Characteristics of the therapists and patients are presented in (Table 1). The therapists had average work experience of 7 years in their current work environment and a mean workload of 77%.

**Table 1:** Participants’ characteristics.

Characteristics	Mean ± SD (Range); n (%)
Patients	N = 15
Age (years)	71.4 ± 15.1 (25–85)
Gender	
Female	9 (60.0%)
Male	6 (40.0%)
Occupation	
Employed	2 (13%)
Retired	12 (80%)
Unspecified	1 (7%)
Infarction type	
Middle cerebral artery stroke	4 (27%)
Ischemic stroke, unspecified	3 (20%)
Basal ganglia stroke	2 (13%)
Pontine stroke	2 (13%)
Thalamic stroke	2 (13%)
Stroke, unspecified	2 (13%)
Therapeutic objective (multiple options)	
Improving gait security / ability to walk	11 (73%)
Living independently at home	8 (53%)
Improving cognitive and memory function, speech	5 (33%)
Improving fine motor skills	4 (27%)
Enhancing activities of daily living	3 (20%)
Strengthening trunk control	2 (13%)
Improving strength in general	1 (7%)
Driving ability	1 (7%)
Vertigo symptoms	1 (7%)
Unspecified	2 (13%)
Therapists	N = 10
Gender	
Female	5 (50%)
Male	5 (50%)

n, number of patients; SD, standard deviation.

### Use of the device

The preparation time to use the device decreased between the first and last session on average from 3 to 1.9 min, while the average calibration time decreased from 5.5 to 4.8 min (mean across all sessions 5.0 min). The average time the device was in use was 15 min across all sessions and all patients. All the available functions of the Holoreach were used. The “stable seat” option was used most frequently during the first session across all patients, while the unlocked mode was the most frequently used during the last session. During the first session the most frequently stated training goal was to “improve functioning of the affected arm”, while “improve trunk control” was the most frequently cited during the last session. The most frequent exercise instruction given was “bring your feet closer together” during the first session, while “lift both feet into the air” was most frequently used during the last session. Two mild and one serious adverse event occurred during the study. The mild events were classified as exhaustion, which is a frequent side effect of rehabilitation. The serious adverse event was due to fainting, without consequential damage, and occurred in a patient who suffered regular fainting attacks outside therapy. It was unrelated to the devices or the study procedures. Data collection for this patient was subsequently terminated, and the patient dropped out of the study. Tables 2 and 3 provide detailed information on usage of the device.

**Table 2:** Usage times.

	Session 1 (n = 15)	Session 2 (n = 14)	Session 3 (n = 14)	Session 4 (n = 9)
Preparation time (minutes) mean ± SD	3.2 ± 1.4	2.9 ± 1.6	2.5 ± 1.1	2 ± 0.6
Calibration time (minutes) mean ± SD	5.5 ± 1.5	5 ± 0	4.9 ± 0.5	4.8 ± 0.7
Exercise time (minutes) mean ± SD	15.3 ± 2.3	15 ± 0	15 ± 0	15 ± 0

n, number of patients; SD, standard deviation.

**Table 3:** Functions used.

	Session 1 (n = 15) n (%)	Session 2 (n = 14) n (%)	Session 3 (n = 14) n (%)	Session 4 (n = 9) n (%)
Degrees of freedom				
Seat locked	10 (67%)	6 (43%)	3 (21%)	1 (11%)
Flexion extension free	2 (13%)	7 (50%)	7 (50%)	4 (44%)
Lateral flexion free	4 (27%)	2 (14%)	5 (36%)	5 (56%)
Completely unlocked	4 (27%)	4 (29%)	7 (50%)	6 (67%)
Training time (in minutes)				
Affected arm	8.1 ± 2.3	6.9 ± 1.4	7.4 ± 0.4	7.0 ± 1.0
Unaffected arm	8.0 ± 4.2	8.1 ± 1.4	7.7 ± 0.4	8.0 ± 1.0
Training goals				
Improve affected arm function	9 (60%)	6 (43%)	4 (29%)	4 (44%)
Improve trunk control	11 (73%)	13 (93%)	13 (93%)	8 (89%)
Exercise instructions				
None	4 (27%)	3 (21%)	3 (21%)	1 (11%)
Put feet closer together	5 (33%)	6 (43%)	7 (50%)	3 (33%)
Lift one foot into the air	3 (20%)	6 (43%)	2 (14%)	1 (11%)
Lift both feet into the air	4 (27%)	8 (57%)	8 (57%)	5 (56%)
I ncrease reaching speed	4 (27%)	3 (21%)	1 (7%)	1 (11%)

multiple answers are possible. N = number of patients.

### Exertion

The median exertion for the lower extremities and trunk increased from the first to the last session (1–3; 3–4.5), while it decreased for the affected arm (3–2) and remained constant for the unaffected arm (2–2). (Table 4).

**Table 4:** Perceived exertion.

	Session 1 (n = 15)	Session 2 (n = 14)	Session 3 (n = 14)	Session 4 (n = 9)
Exertion for the legs (0 = not at all exhausting, 10 = very exhausting)				
Median (Range)	1 (0–6)	2 (0–6)	3 (0–6)	3 (1–6)
Exertion for the trunk (0 = not at all exhausting, 10 = very exhausting)				
Median (Range)	3 (0–6)	2.5 (0–7)	2 (1–6)	4.5 (0–6)
Exertion for the affected arm (0 = not at all exhausting, 10 = very exhausting)				
Median (Range)	3 (0–8)	1.5 (0–9)	3 (0–5)	2 (1–5)
Exertion for the unaffected arm (0 = not at all exhausting, 10 = very exhausting)				
Median (Range)	2 (0–5)	2.5 (0–4)	1 (0–5)	2 (1–5)

n = number of patients; SD = standard deviation.

### Themes

The following main themes were investigated: (1) Expectations on training with Holoreach, (2) Overall impressions, (3) Preparation and conduction of training, (4) Level of challenge, (5) Perceived training effectiveness, (6) Motivation, (7) Safety, (8) Target group and (9) Extra time training. The exercise time for the affected arm decreased from an average of 8 min during the first session to 7 min during the last session, while it remained constant for the unaffected arm at 8 min. The most frequent exercise goal during the first session was “function improvement of the affected arm”, while “trunk control” was the most frequent exercise goal during the last session. Through the questionnaires both therapists and patients gave overall positive feedback on the Holoreach across all themes. However, between 9 and 40% of the therapists and 0% and 13% of the patients did not respond to individual questions (for two particular questions this was 60% and 87%).

#### Expectations on training with Holoreach

During the focus group discussions, all the therapists expressed excitement about the novel training tool and curiosity as to how patients would react to it. The expectation was that the Holoreach would be welcomed during repetitive exercises due to its motivating effect. At the same time, they expressed skepticism about: the limited field of view provided by the MHL, the preparation time required, and possible adverse events such as dizziness.

#### Overall impression

During the focus group meetings, the therapists expressed overall enthusiasm for the device. They characterized it as new, motivating, fresh, joyful, interesting, and exciting. They lauded its perceived user friendliness, the quick setup, the visuals, and playfulness of the device. Furthermore, they commended the opportunities provided by the mobile seat regarding increased variety of exercise and its anticipated effect on trunk control.

#### Preparation and conduction of training

**Figure 2:**
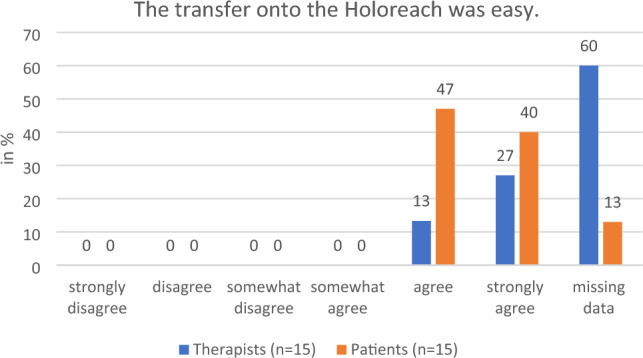
Therapists’ (n = 15) and patients’ (n = 15) evaluation of the question on whether transfer onto the Holoreach was easy.

**Figure 3:**
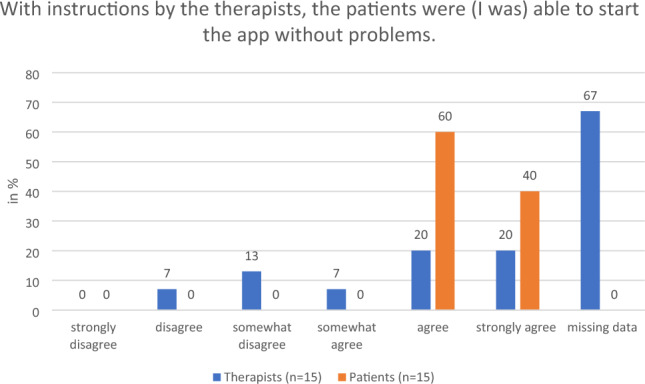
Therapists’ (n = 15) and patients’ (n = 15) evaluation of the question about whether the patients were (I was) able to start the app without problems.

**Figure 4:**
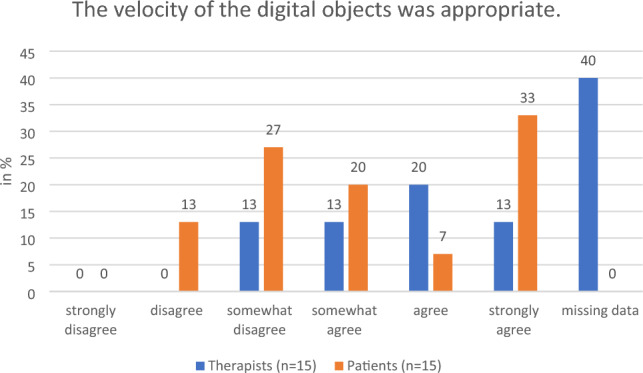
Therapists’ (n = 15) and patients’ (n = 15) evaluation of the question about whether the velocity of the digital objects was appropriate.

During the focus group discussions, the therapists regarded the preparation as largely problem-free and commented favorably on the preparation time. They praised the adjustability of the chair height and armrests since they ease the transfer of patients who require assistance into the chair. They were also positive about ease of use of the Holoreach: “Put it on, turn it on and off we go.” The option to work with a desktop PC screen was appreciated. These findings were supported by the questionnaire responses of both therapists and patients. For example, 100% of all participants who answered the questions said that they either “strongly agreed” or “agreed” with the statement that the transfer onto the Holoreach was easy to conduct and that after calibration the patients still had enough energy to perform the exercises (Fig. 2). There was a discrepancy between the impressions of the therapists and patients regarding the patient’s ability to start the app independently. While 100% of patients stated that they “strongly agreed” or “agreed” with the statement that when following instructions from the therapists, they were able to start the app without problems, 20% of therapists either “disagreed” or “somewhat disagreed” with the same statement (Fig. 3). The therapists commented on the position of the digital objects in the patient’s field of view and recommended that they should appear no lower than the patient’s stomach and not too close to the patient’s body. They suggested that the therapists need more options for influencing the position of the objects, the velocity with which they move, and the ability to pause the game. Of the patients 40% and of the therapists 13% “disagreed” or “somewhat disagreed” with the statement that the velocity of the digital objects was appropriate (Fig. 4). Furthermore, 34% of patients and 13% of the therapists “disagreed” or “somewhat disagreed” with the statement that the distance of the digital objects was appropriate.

#### Level of challenge

**Figure 5:**
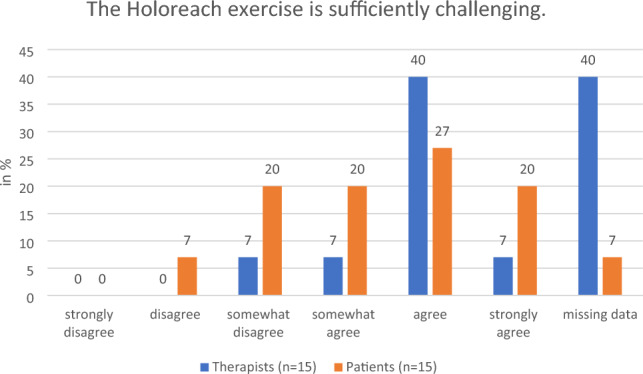
Therapists’ (n = 15) and patients’ (n = 15) evaluation of the question of whether the Holoreach exercise is sufficiently challenging.

All therapists appreciated the option to adjust the level of challenge individually for a patient using the unlocked mode of the dynamic seat and the instructions on adjusting the position of the patient’s feet. They suggested including options for the therapist to change the velocity of the digital objects, their starting position, and trajectories. In addition, intermediate states between a locked and an unlocked seat were suggested as useful. They commended the range of motion of the dynamic seat. Agreement with these findings was underlined by the 7% of the therapists and 27% of patients who responded with “somewhat disagree” or “disagree” to the statement “The Holoreach exercise is sufficiently challenging” (Fig. 5).

#### Perceived effectiveness of the training

**Figure 6:**
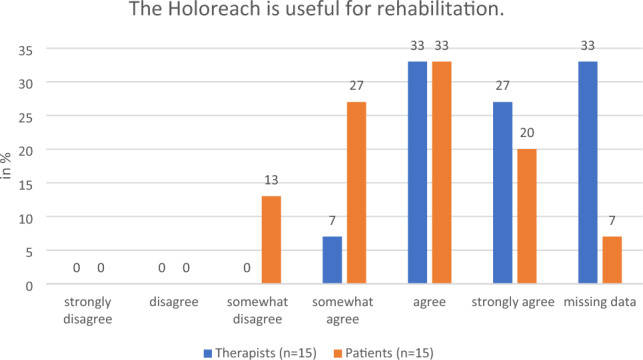
Therapists’ (n = 15) and patients’ (n = 15) evaluation of the question whether the Holoreach is useful for rehabilitation.

**Figure 7:**
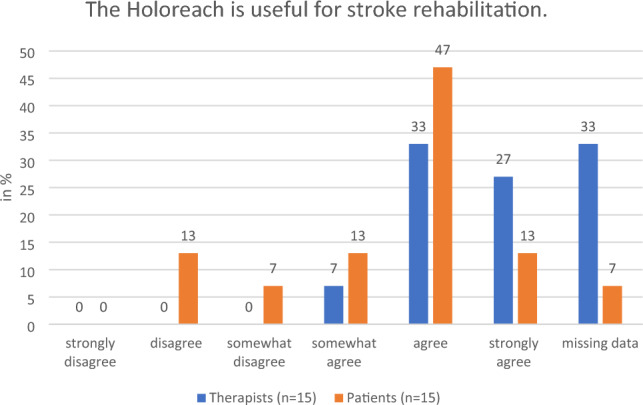
Therapists’ (n = 15) and patients’ (n = 15) evaluation of the question whether the Holoreach is useful for stroke rehabilitation.

**Figure 8:**
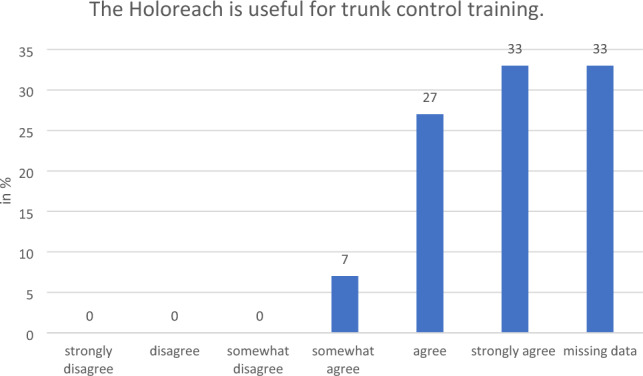
Therapists’ (n = 15) answers to the question on usefulness of the Holoreach for trunk control training.

**Figure 9:**
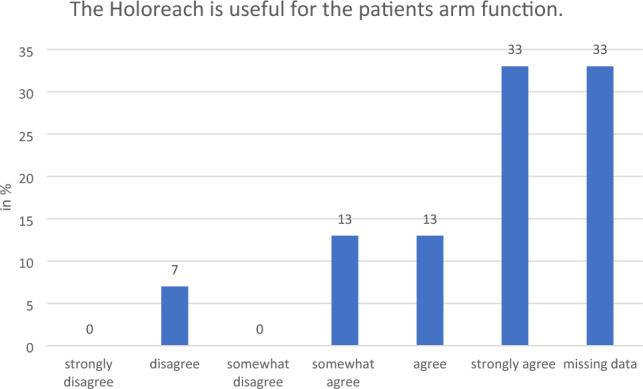
Therapists’ (n = 15) evaluation of the question on the usefulness of the Holoreach for patients’ arm function.

All the therapists who responded and 80% of patients said that they would “strongly agree”, “agree” or “somewhat agree” with the statement that training with a Holoreach is beneficial for rehabilitation (Fig. 6). Moreover, all the therapists who responded and 73% of patients said that they also would “strongly agree”, “agree” or “somewhat agree”, with the statement that training with a Holoreach is beneficial for stroke rehabilitation (Fig. 7). Likewise, all therapists stated that they “strongly agree”, “agree” or “somewhat agree” that training with Holoreach is useful for the patients arm function, and only 7% “disagreed” that it is also useful for trunk control training (Figs. 8, 9). Sixty-seven % of patients stated that they “strongly agree”, “agree” or “somewhat agree” that training with Holoreach has variable results.

#### Motivation

**Figure 10:**
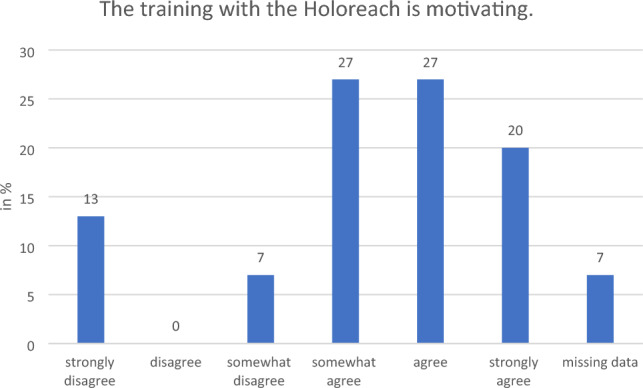
Therapists’ (n = 15) evaluation of the question on whether training with the Holoreach is motivating.

During the focus group meetings, the therapists regarded Holoreach as motivating due to the adaptable level of challenge but also gave suggestions on how motivation can be kept high during rehabilitation. They had several recommendations to improve motivation. To maintain and improve motivation and the transfer to daily activities, the therapists recommended a greater variety of digital objects, exercises that involve a selection of digital objects to increase the cognitive challenge, exercises that include gripping and placing of objects, other ADL activities such as cutting objects or painting walls, variable auditory feedback, awards, and bimanual tasks. The seat and the MHL could be connected so that both elements react to the progression of the training or to a situational overload of the patient, thus fine tuning the difficulty level. This is underlined by the patients’ responses to the question whether training with Holoreach is motivating: While 74% responded that they would either “strongly agree”, “agree” or “somewhat agree” with this statement, 7% “somewhat disagreed” and 13% “strongly disagreed” (Fig. 10).

#### Safety

**Figure 11:**
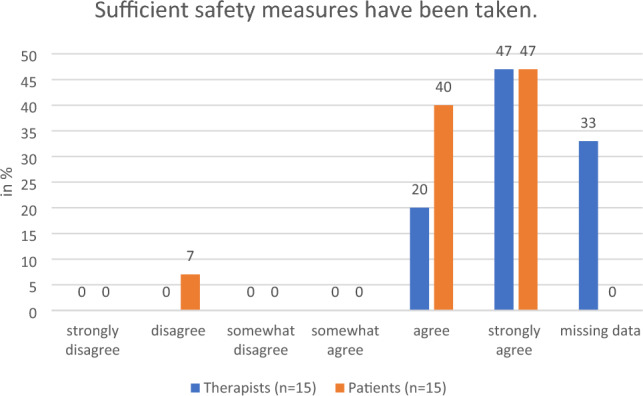
Therapists’ (n = 15) and patients’ (n = 15) evaluation of the question on whether sufficient safety measures have been taken.

The therapists regarded the prototype Holoreach as safe and gave suggestions for future iterations. To avoid adverse events (see “Use of the device” section) the chest strap should be adjusted. The belt was quite restrictive for some patients, while patients with a small body size could not be strapped tightly enough. To accommodate this the strap needs to be lower or the seat surface higher. Confidence in safety of the Holoreach was underlined by the responses to the questionnaire: 67% of the therapists agreed that sufficient safety measures had been taken and 87% of the patients agreed that they felt safe during training with the Holoreach. Regarding the serious adverse event, no direct connection to the device could be established. While a possible connection between a specific head movement (head tilted forward) and the serious adverse event cannot be ruled out with certainty it is unlikely. Such activities (head tilted forward) are very common in a patient’s daily therapy setting and everyday life and do not only occur in this study (e.g., eating) (Fig. 11).

#### Target group

The therapists found the Holoreach to be suitable for patients who can sit stably, follow and understand instructions. Furthermore, the Holoreach seems suitable for patients with severe walking impairments as reaching movements required when sitting are trained, which is useful for everyday life. Following clinical examination, the therapists should judge whether training with the Holoreach is appropriate for a particular patient. Defining this solely on an assessment scale is considered difficult.

#### Potential for extra time training

While the therapists appreciate more options for spending extra time on training, they also expressed some reservations about the potential of Holoreach for spending extra time on training. During the study, patients needed support in starting and operating the software, as well as for putting on and taking off the MHL and attaching the safety straps.

## Discussion

This study used a UCD approach to assess the usability of the prototype Holoreach system for the rehabilitation of trunk control and arm function of patients after stroke. The Holoreach represents an advance at various levels in rapidly evolving neurorehabilitation, particularly regarding practical points of exercise delivery^43^. The Holoreach integrates a streamlined calibration module and an exercise module that provide clinically relevant and functional exercise without requiring the patient to change position during the exercise or requiring much physical space^43–45^. The low footprint impact on premises allows for a functional, quiet, and professional therapy environment and almost barrier-free interaction between therapist and patient^46^. Since no further exercise materials are required, the complexity of the exercises remains low, which is beneficial for implementation into the rehabilitation continuum^43^. This aspect could also make the system suitable for telerehabilitation settings in the future^47^. Motivation is important^48^ and the MR system can support therapists in the use of motivational strategies such as practicing with gaming properties, and present progress in a way that confirms the information of the therapy sessions^49^ with the long-term aim of maintaining or increasing adherence. The mobile seat component, when combined with visual feedback and gamification via a flat screen, has been shown to be a useful technology for selective trunk control exercises that result in clinically relevant improvement in trunk control, contrary to a control group receiving standard care^23^. Generally, the prototype Holoreach was found to be a device suitable for training the trunk and arm control for post stroke patients.

The improvement suggestions below are sub-grouped into hardware, software, and design requirements for ease of reading.

### Hardware requirements

The level of challenge could be made more customizable by linking the seat motion with the MHL thereby allowing more influence on the range of motion and trunk control during the reaching motions. The MHL’s field of view is limited due to the small projection area of the lens. Consequently, objects are only detected if the eye line of vision corresponds with the approximate position of the object in space in front of the lens. This results in the need for patients to generate substantial head and trunk movements to detect objects. A lens with an enhanced field of view would enable the identification of objects by eye movement and could reduce an excessive amount and head and trunk movements for patients for whom this is necessary due to impairments. It would be useful if the chest strap could be better adapted to the movements of the torso so as not to hinder the patients during the exercise. For this, straps that are flexible could be a solution as they allow a substantial range of motion but still provide adequate safety, preventing the patient from falling forward.

### Software requirements

Exercises tailored to the needs of the users more accurately than is currently the case with this prototype should be included to allow a greater variety of exercise and level of difficulty. A greater range of exercises could be achieved by including exercises that are based on neural coupling, or error augmentation^50–52^. Exercises that involve the bilateral hand movements common to ADL would be useful. These might involve holding and cutting digital objects, which would support activation of the neural coupling mechanism^53^. However, it remains unclear if, in absence of afferent cutaneous sensory information of the hands, visual information on hand position can support improvements in ADL activities^54^. Elements of visual distortion, such as shrinking or bending the digital objects, could be added to support motor learning by making use of error augmentation principles^55,56^. Nevertheless, further research is necessary on the transfer, and thus usefulness, of a possible kinematic adaptation process to real everyday function involving kinetic masses^57–59^. The anticipated variations in difficulty and progressive elements could be provided through faster moving objects following non-linear trajectories, or exercises in which cognitive tasks are included (e.g., only objects of a certain color may be caught). Additionally, separate treatment of the affected and less affected arm would support an adjusted therapy sequence appropriate for the current impairment level. The controller unit might be enhanced by incorporating psychophysiological measurements to better adjust difficulty, progression, and duration of the exercises^60^. For example, by integrating eye trackers to detect the mental fatigue of stroke patients, should this approach prove feasible in a stroke population^61,62^. A more effective adaption of the control software would be to give both patients and therapists the option of manually adjusting parameters such as the speed, position, composition, or number of objects, thus handing them direct control over the difficulty level and exercise progression. This suggestion is supported by findings that utilize opinions of stroke patients as ground truth data for classifying exercise difficulties. Using the patient’s opinion as ground truth data was rated as superior to the therapist’s opinion, with or without the addition of psychophysiological, or movement data^63^.

### Design requirements

Kinematic and kinetic data measured by the prototype may provide interesting insights in the performance of the patients and assessment of trunk stability. Sensors to identify stabilization skills of patients may track the amplitudes of the mobile seat due to upper body deflection during reaching. The hand position can be tracked in the MHL software. These synchronized data could in turn support a tailored adjustment of difficulty level regarding speed of obstacles and position. The addition of a pressure mat on the seat to identify Centre of Pressure (CoP) could be beneficial in the assessment and also for feedback to patients^64^.

A previous iteration of the device included a motorized mode with unidirectional (flexion–extension, lateral flexion) or bilateral (circle, Fig. of 8) actuated movements of the mobile seat. Reintegration might support patients in relearning optimization strategies for trunk control during reaching movements with a further level of difficulty of a moving surface^65^. A motorized version with faster actuation could be used to generate sudden perturbations that would allow for disturbances that impede goal achievement during exercises, and thus provide a modality to train impedance control of the trunk and the extremities^66^. Another aspect that could increase motivation is the introduction of a cooperative mode with visual and auditory communication between two or more patients where a therapist adapts the settings individually^67^. This approach has been shown to be feasible as well as motivating when utilizing a treadmill-virtual reality system for home training of two patients with Parkinson disease^68^.

All further alterations resulting from the suggestions would need to be tested in an agile development process, with each alteration being analyzed, the deficits defined, and the design adapted to improve the device. In this manner, new requirements would be identified during the process^69^.

### Outlook

The use of UCD has shown that the involvement of potential users in the early design and testing of a technology is useful in specifying requirements and in promoting the intended health outcomes^70^. Limitations thus identified need to be improved through agile development processes and more intensive technological development of the hardware and software. We can only analyze the statements of people who completed the questionnaires. People who did not complete the questionnaire are not included in the results (possibly resulting in a selection bias). Future research on this novel therapy approach should also address its effectiveness in rehabilitation of trunk control and arm function in a larger trial than in previous iterations^23^. Furthermore, its mode of application—which setting is useful as a complement to physical and occupational therapies (e.g., in-house, outpatient or telerehabilitation) needs further evaluation.

User involvement is crucial in technology development, with models such as UCD and participatory design (PD) emphasizing the incorporation of user needs throughout the development process. PD, commencing earlier than UCD, places a distinct focus on the fundamentals of users’ roles, considering them as partners who actively contribute to content creation and decision-making^71,72^. Additionally, objective measurements of usability will be employed during subsequent development steps.

## Conclusions

Holoreach is a promising and novel therapy approach that enhances exercise opportunities for post stroke patients. It has the potential to be an add-on for the treatment of trunk control and arm function. Improvements in the hardware and software are necessary, to better personalize the exercises and the level of difficulty, and to understand the movement and balance strategies of patients, towards improving Holoreach to be capable of functioning as a counterpart to regular physical therapy treatment.

### Supplementary Information

Supplementary Information.

## Data Availability

The datasets used and/or analyzed during the current study are available from the corresponding author on reasonable request.
