# Supplementary material for: Therapists and patients perceptions of a mixed reality system designed to improve trunk control and upper extremity function
Source: Sci Rep. 2024 Mar 19;14:6598. doi: 10.1038/s41598-024-55692-4 (PMC10951291; doi:10.1038/s41598-024-55692-4)
Supplement: Supplementary file 1 — Supplementary Information. [file 41598_2024_55692_MOESM1_ESM.docx]

## Participant information

| Name, Surname: |  |
| --- | --- |
| I am a … | **□** Physiotherapist |
|  | **□** Occupational Therapist |

***Information about the patient:***

| Age: |  | |  |  |  |  |
| --- | --- | --- | --- | --- | --- | --- |
| Gender: | **□** | | male | **□** | female |  |
| Occupation: | |  | |  |  |  |
| Diagnosis: |  | |  |  |  |  |
| Nature and Duration of complaints: | | | | |  |  |
|  |  | |  |  |  |  |
| Therapy goal: | | | | | | |
|  |  | |  |  |  |  |

## Study Diary

**Please complete following the training with Holoreach.**

Please fill out a diary especially when you have done other exercises or new problems/difficulties have arisen.

*Additional diary forms can be filled out if needed (you will receive some forms in reserve. If these are not sufficient, please request more at mandy.scheemesser@zhaw.ch).*

Diary 1

***To be completed by the therapist(s).***

**Date: ________**

**Preparation time[min]: ________**

**Calibration time [min]: _______**

**Training time [min]: ________**

| Which settings of the seat did you use during the exercises? (Multiple answers possible)  □ Locked  □ anterior/posterior free  □ mediolateral free  □ Unlocked | | | |
| --- | --- | --- | --- |
| With which arm and for how long did your patient perform the exercises? | | | |
| □ Affected Arm: Training time: ___ min | | | |
| □ Unaffected Arm: Training time: ___ min | | | |
| What were the exercise goals? (Multiple answers possible)  □ Functional improvements of the affected arm  □ Improvement of trunk control  □ Others: _____ | | | |
| What instructions did you give to increase the difficulty of the exercises? (Multiple answers possible) | | | |
| □ none  □ Put feet closer together  □ Lift one feet up into the air  □ Lift both feet up into the air  □ Grasp objects faster (Increase grasp frequency)  □ Others: | | | |
| Please give reasons if the exercise session of 15 minutes had to end early? (Multiple answers possible) | | | |
| □ Patient was exhausted  □ Patient lost motivation  □ Battery down  □ others: | | | |
| What score did the patient achieve in the exercise session?  _________ | | | |
| **Were there any problems or difficulties during the therapy with the Holoreach?** | | | |
| □ | No |  |  |
| □ | Yes → which? |  |  |

***To be completed by the patient:***

| **Exertion:** How strenuous were these exercises for your legs, trunk, and arms? | | | | | | | | |
| --- | --- | --- | --- | --- | --- | --- | --- | --- |
| **Legs** | | **Trunk** | | **Affected arm** | | **Unaffected arm** | |  |
| **□** | **0** | **□** | **0** | **□** | **0** | **□** | **0** | **Not strenuous at all** |
| **□** | **1** | **□** | **1** | **□** | **1** | **□** | **1** |  |
| **□** | **2** | **□** | **2** | **□** | **2** | **□** | **2** |  |
| **□** | **3** | **□** | **3** | **□** | **3** | **□** | **3** |  |
| **□** | **4** | **□** | **4** | **□** | **4** | **□** | **4** |  |
| **□** | **5** | **□** | **5** | **□** | **5** | **□** | **5** |  |
| **□** | **6** | **□** | **6** | **□** | **6** | **□** | **6** |  |
| **□** | **7** | **□** | **7** | **□** | **7** | **□** | **7** |  |
| **□** | **8** | **□** | **8** | **□** | **8** | **□** | **8** |  |
| **□** | **9** | **□** | **9** | **□** | **9** | **□** | **9** |  |
| **□** | **10** | **□** | **10** | **□** | **10** | **□** | **10** | **Very strenous** |

| **Discomfort:** If you have experienced pressure sores, injuries, pain, dizziness, or other discomfort during therapy? | | | |
| --- | --- | --- | --- |
| □ | No |  |  |
| □ | Yes → Where have which complaints occurred? |  |  |

## Therapists’ questionnaire

*To be completed at the end of the study (1 per patient).* ***Date: ___________***

1. Please evaluate the following statements.

|  | | | **Strongly disagree** | **disagree** | | | **Somewhat disagree** | **Somewhat agree** | | **agree** | **Strongly agree** | | |
| --- | --- | --- | --- | --- | --- | --- | --- | --- | --- | --- | --- | --- | --- |
| **Preparation** | | | **1** | **2** | | | **3** | **4** | | **5** | **6** | | |
| *If the patient uses a wheelchair (otherwise leave empty):* The transfer from the wheelchair to the Holoreach chair was easy to perform. | | | **□** | **□** | | | **□** | **□** | | **□** | **□** | | |
| The seat height of the chair was easy to adjust. | | | **□** | **□** | | | **□** | **□** | | **□** | **□** | | |
| The backrest was easy to adjust. | | | **□** | **□** | | | **□** | **□** | | **□** | **□** | | |
| The chest belt was easy to adjust. | | | **□** | **□** | | | **□** | **□** | | **□** | **□** | | |
| The armrests made it easier for the patient to sit on the chair. | | | **□** | **□** | | | **□** | **□** | | **□** | **□** | | |
| The patient was able to start the app with my instructions without any problems. | | | **□** | **□** | | | **□** | **□** | | **□** | **□** | | |
| With the help of the fact sheet the Holoreach could be operated without any problems. | **□** | | | | **□** | | **□** | | **□** | **□** | **□** | | |
| The time spent on preparations was reasonable. | | | **□** | **□** | | | **□** | **□** | | **□** | **□** | | |
| **Calibration** | | **1** | | | **2** | | **3** | | **4** | **5** | **6** | | |
| The patient was able to start the calibration without any problems. | | **□** | | | **□** | | **□** | | **□** | **□** | **□** | | |
| The calibration was trouble-free. | | **□** | | | **□** | | **□** | | **□** | **□** | **□** | | |
| The patient was able to perform the movements required for calibration without any problems. | | **□** | | | **□** | | **□** | | **□** | **□** | **□** | | |
| The patient still had enough energy after calibration to start the exercise as well. | | **□** | | | **□** | | **□** | | **□** | **□** | **□** | | |
| The patient was able to block out non-essential visual stimuli well. | | **□** | | | **□** | | **□** | | **□** | **□** | **□** | | |
|  | | | **Strongly disagree** | **disagree** | | | **Somewhat disagree** | **Somewhat agree** | | **agree** | **Strongly agree** | | |
| **Exercise** | | | **1** | **2** | | | **3** | **4** | | **5** | **6** | | |
| The static objects were easily recognizable for the patient. | | **□** | | | **□** | | **□** | | **□** | **□** | **□** | | |
| The dynamic objects (arrows, floating fruits) were easily recognizable for the patient. | | **□** | | | **□** | | **□** | | **□** | **□** | **□** | | |
| The speed of the dynamic objects was appropriate for the patient. | | **□** | | | **□** | | **□** | | **□** | **□** | **□** | | |
| The distance to the objects to be reached was appropriate for the patient. | | **□** | | | **□** | | **□** | | **□** | **□** | **□** | | |
| The exercise was sufficiently challenging for the patient. | | **□** | | | **□** | | **□** | | **□** | **□** | **□** | | |
| The exercise did not overwhelm the patient. | | **□** | | | **□** | | **□** | | **□** | **□** | **□** | | |
| **Overall training (Calibration and exercise combined)** | **1** | | | | **2** | | **3** | | **4** | **5** | **6** | | |
| Training with Holoreach is useful for the rehabilitation of patients after a stroke. | **□** | | | | **□** | | **□** | | **□** | **□** | **□** | | |
| Training with Holoreach is useful for the rehabilitation of this patient. | **□** | | | | **□** | | **□** | | **□** | **□** | **□** | | |
| Training with Holoreach is useful for training trunk control of this patient. | **□** | | | | **□** | | **□** | | **□** | **□** | **□** | | |
| Training with Holoreach is useful for training this patient's arm function. | **□** | | | | **□** | | **□** | | **□** | **□** | **□** | | |
| **Safety** | **1** | | | | **2** | **3** | | | **4** | **5** | | **6** |  |
| The backrest provides sufficient safety for the patient. | **□** | | | | **□** | **□** | | | **□** | **□** | | **□** |  |
| The armrests provide sufficient safety for the patient when sitting down and standing up. | **□** | | | | **□** | **□** | | | **□** | **□** | | **□** |  |
| The chest belt provides sufficient safety for the patient. | **□** | | | | **□** | **□** | | | **□** | **□** | | **□** |  |
| Sufficient safety measures have been taken. | **□** | | | | **□** | **□** | | | **□** | **□** | | **□** |  |
| 1. If you could not fully agree with a statement: Please briefly describe the problems or difficulties. | | | | | | | | | | | | |  |
|  | | | | | | | | | | | | |  |
| 1. What is your overall impression of Holoreach? | | | | | | | | | | | | |  |
|  | | | | | | | | | | | | |  |
| 1. Which aspects would you like to discuss in more depth in the focus group? | | | | | | | | | | | | |  |
|  | | | | | | | | | | | | |  |

## Patients Questionnaire

*To be completed at the end of the study.* ***Date: ______________***

1. Please evaluate the following statements.

|  | | **Totally disagree** | | | **disagree** | **Rather disagree** | **Rather agree** | | **agree** | **Totally agree** |
| --- | --- | --- | --- | --- | --- | --- | --- | --- | --- | --- |
| **Preparation** | | **1** | | | **2** | **3** | **4** | | **5** | **6** |
| With the support of the therapist, I was able to sit well in the chair of Holoreach. | | **□** | | | **□** | **□** | **□** | | **□** | **□** |
| The armrests made it easier for me to take a seat on the chair. | | **□** | | | **□** | **□** | **□** | | **□** | **□** |
| The seat height of the chair was easy to adjust to my needs. | | **□** | | | **□** | **□** | **□** | | **□** | **□** |
| The backrest was easy to adjust to my needs. | | **□** | | | **□** | **□** | **□** | | **□** | **□** |
| The chest belt was comfortable. | | **□** | | | **□** | **□** | **□** | | **□** | **□** |
| With the guidance of my therapist(s) I was able to launch the app without any problems. | | **□** | | | **□** | **□** | **□** | | **□** | **□** |
| The time required for the preparations was reasonable. | | **□** | | | **□** | **□** | **□** | | **□** | **□** |
| **Calibration** | | **1** | | | **2** | **3** | **4** | | **5** | **6** |
| With the guidance of my therapist(s) I was able to start the calibration without any problems. | | **□** | | | **□** | **□** | **□** | | **□** | **□** |
| The calibration ran without any problems. | | **□** | | | **□** | **□** | **□** | | **□** | **□** |
| I was able to perform the movements necessary for calibration without any problems. | | **□** | | | **□** | **□** | **□** | | **□** | **□** |
| I still had enough energy after the calibration to start the exercise as well. | | **□** | | | **□** | **□** | **□** | | **□** | **□** |
| I was able to concentrate well on picking the rows of fruit. | | **□** | | | **□** | **□** | **□** | | **□** | **□** |
|  | | | **Totally disagree** | **disagree** | | **Rather disagree** | **Rather agree** | | **agree** | **Totally agree** |
| **Exercise** | | | **1** | **2** | | **3** | **4** | | **5** | **6** |
| I could see the static objects (fruits) well. | | **□** | | | **□** | **□** | | **□** | **□** | **□** |
| I could see the dynamic objects (arrows, floating fruits) well. | | **□** | | | **□** | **□** | | **□** | **□** | **□** |
| The speed of the dynamic objects was appropriate. | | **□** | | | **□** | **□** | | **□** | **□** | **□** |
| The distance of the fruit to be grabbed was appropriate. | | **□** | | | **□** | **□** | | **□** | **□** | **□** |
| The exercise with Holoreach was sufficiently challenging for me. | | **□** | | | **□** | **□** | | **□** | **□** | **□** |
| The exercise with Holoreach did not overwhelm me. | | **□** | | | **□** | **□** | | **□** | **□** | **□** |
| I could concentrate well on picking the fruit. | | **□** | | | **□** | **□** | **□** | | **□** | **□** |
| **Overall training (Calibration and exercise combined)** | **1** | | | | **2** | **3** | | **4** | **5** | **6** |
| The training with Holoreach was useful for my rehabilitation. | **□** | | | | **□** | **□** | | **□** | **□** | **□** |
| The training with Holoreach was varied. | **□** | | | | **□** | **□** | | **□** | **□** | **□** |
| The training with Holoreach was motivating. | **□** | | | | **□** | **□** | | **□** | **□** | **□** |
| The score at the end of the exercise motivated me to try hard. |  | | | |  |  | |  |  |  |
| Training with Holoreach is useful for the rehabilitation of patients after a stroke. | **□** | | | | **□** | **□** | | **□** | **□** | **□** |

| **Safety** | **1** | **2** | **3** | **4** | **5** | **6** |
| --- | --- | --- | --- | --- | --- | --- |
| The backrest provided me with enough security. | **□** | **□** | **□** | **□** | **□** | **□** |
| The armrests provided me with enough security to sit on the chair. | **□** | **□** | **□** | **□** | **□** | **□** |
| The chest belt offered me enough security. | **□** | **□** | **□** | **□** | **□** | **□** |
| During training with Holoreach I felt safe. | **□** | **□** | **□** | **□** | **□** | **□** |

| 1. If you could not fully agree with a statement: Please briefly describe the problems or difficulties. |
| --- |
|  |
| 1. What is your overall impression of Holoreach? |
|  |

## Holoreach: Guide for the focus group discussion

**Place: Date: Time:**

**Participants:**

**Introduction:**

- Introducing the moderator
  - Person
  - Role: leads discussion, but does not participate with own views, asks if something is unclear
- Role of the developer
  - Introduces himself.
  - Role: Was involved in the development of Holoreach, knows how it works and can ask specific questions if feedback is unclear

**Aim and framework of the group discussion:**

- Aim of the user study: use of Holoreach (prototype rehabilitation device for trunk control, consisting of a chair with a movable seat and a Microsoft Hololens 2.0, a pair of glasses with integrated games (mixed reality glasses) in the rehabilitation of patients with impaired balance after a stroke.
- Aim of the focus group - collecting therapists' experiences with the Holoreach so far; what is going well, what is there to improve? The possibilities for improvement are particularly important to us.
- All views and opinions are relevant - not necessarily agreement sought, but controversy desired.
- All can raise questions; answers are also to be understood as further food for thought.
- Confidentiality guaranteed - responses are summarized and anonymized.
- Duration (estimate): 1h – 1.5h
- Recording on audio carrier for repeated listening, relieves the burden of notetaking.
- Declaration of consent.

| **Topic** | **Main Question** | **Follow Up Questions / Possible Answers** |
| --- | --- | --- |
| Introduction | Please introduce yourself briefly | - Number of years in the profession, workload, which patients do you treat? |
| General | How did you treat your stroke patients until now? |  |
|  | What is your general impression on Holoreach? | - What expectations did you have regarding Holoreach? - Have those been fulfilled? - Which expectations were fulfilled, which not? - Which attributes of Holoreach did you like, which didn’t you like? - What types of difficulties did you encounter? |
| Preparation | How did you cope with the start-up of the Holoreach (transfer to the chair, guiding the patient to put on the glasses and start the Hololens)? | - How well can the Holoreach stool be adapted to the patient, e.g., body size? - Are there any setting options that you were missing but that you would like to have considered in a further development? |
|  | In your opinion, how did the patients cope with the start-up of the Holoreach (transfer to the chair, putting on the glasses and starting the Hololens)? |  |
|  | In your opinion, what should be improved in the preparation? |  |
|  | How did you and the patients cope with the calibration of the Hololens? | - How well did the patient manage to start the calibration? - How well did the patient manage to perform the movements necessary for calibration? |
| Operation/functionality | Were there any technical problems with the Holoreach during the therapy? | - How did you deal with it? - How did the patients deal with it? |
|  | Have you used the factsheet? | - How helpful did you find the factsheet? - Did you miss any information? |
|  | How well were the patients able to use the software? | - Were there any problems? If yes, which ones? |
|  | To what extent do you think it is conceivable that patients will be able to use the Holoreach independently? |  |
|  | For which impairment level/patient condition is the use of the Holoreach ideal? |  |
|  | In your view, are certain functions missing from the Holo-reach? |  |
| Therapy | How well can the Holoreach be adapted to the patient's abilities/needs? | - Were the patients challenged but not overwhelmed in their range of motion? |
|  | How often did you use the different locking and unlocking modes? | - Under what circumstances did you have patients exercise under the following conditions:   - Locked   - Anterior/posterior free   - Mediolateral free   - Unlocked |
|  | How well were the patients able to reach for the fruit? | - For static fruits - For dynamic fruit - How helpful do you consider the green arrows indicating where the next fruit appears? - How well were patients able to concentrate on picking the fruit? - Were there any distractions? |
|  | How often were patients able to complete the exercises? | - If they could not complete it: for what reason? - What should be changed in the therapies of the Holoreach, so that the patients can train/practice better/successfully? |
|  | Could you imagine further exercises? If so, which ones specifically? |  |
|  | For which other patient groups could the Holoreach be used? | - What would need to be considered? |
|  | What requirements would patients have to meet to be able to train (independently) with the Holoreach**?** |  |
| Safety | How do you assess the safety of the Holoreach for different patients? | - Do the belts, the backrest, and the armrests provide the necessary support? - Does it need a headrest or footrest? |
|  | Were there any (unexpected) incidents during the therapy? | - If so, please describe them. - How did you deal with them? |
| Conclusion | What advantages and disadvantages do you see in the Holoreach compared to conventional training of stroke patients? | - How useful do you find the Holoreach...   - for training trunk control?   - for training arm function? |
|  | Potential for improvement: What are the most important requirements for the further development of Holoreach? | - How would you prioritize these requirements? |
|  | Are there any topics that have not yet been addressed? |  |
